# Supplementary material for: Deep learning-based correction of cataract-induced influence on macular pigment optical density measurement by autofluorescence spectroscopy
Source: PLoS One. 2024 Feb 13;19(2):e0298132. doi: 10.1371/journal.pone.0298132 (PMC10863874; doi:10.1371/journal.pone.0298132)
Supplement: Attachment — Submitted filename: renamed_8986a.docx [file pone.0298132.s003.docx]

Reply to reviewers.

Our reply is written in blue font.

Dear Reviewer #1

We appreciate your review work and thanks for your very useful comments and suggestions on improving our manuscript. We investigated each suggestion carefully and revised our manuscript accordingly. Our reply is written in blue font. The revised parts were highlighted with red font in the revised manuscript.

Reviewer #1: The authors reported the efficacy of deep learning-based correction of cataract-induced errors of macular pigment optical density (MPOD) calculated by the subtraction of autofluorescence on a fluorescein angiography device. As the authors stated, MPOD measurement is important to investigate impacts of MPOD on development and progression of age-related macular degeneration. Cataract interferes with capture of high quality images of fundus autofluorescence. They insisted on usefulness of deep learning to correct measured values. The manuscript was succinctly written. However, there may be some issues to be addressed. First of all, it may be necessary to explain the impacts of cataract and intraocular lens on autofluorescence imaging so that general readers can understand the content easily. Although the authors stated that cataract interferes excitation light transmission, it may be also critical that cataract itself has autofluorescence, increasing background density of captured images. The authors should discuss this issue. Also, there are two types of intraocular lens (IOL): clear and yellow-tinted. The latter may block somewhat blue light transmission, potentially affecting captured images of autofluorescence. Did all patients undergo the implantation of the same type IOL? It would be better to provide representative images before and after cataract surgery. Second, it would be better to provide graphs to show the relationship of age and MPOD before and after surgery and that of cataract status and MPOD.

- Thank you very much for your extremely helpful suggestions. Given that the readers of this journal include individuals beyond the field of ophthalmology, we have added a note at line 103 highlighting that the measurement methods for macular pigments are not yet fully established clinically. Additionally, I have made an annotation regarding the measurement methods at line 121.
- We have revised the description to make it more understandable for readers outside the field of ophthalmology regarding the impact of cataracts (line 130-147). Additionally, we have added information regarding the autofluorescence of the cataract lens (line 140).
- All patients received implantation of yellow-tinted intraocular lens. We described it in line 192. The measurement of MPOD by autofluorescence spectroscopy is considered not to be affected by the transmission rate of the IOL theoretically because MPOD is calculated based on the ratio of blue light blockage between the targeted lesion and background lesion (8.98 degree) and both lesions are affected equally by the IOL. Strictly, the difference in the thickness of IOL between the center of the lens and peripheral region of the lens may influence the measurement, but the difference was considered negligible.

(Lines 2, 56) The term, ‘autofluorescence technique’ should be ‘the autofluorescence subtraction method’.

- Thank you for your suggestion. We changed the term “autofluorescence technique” to “autofluorescence spectroscopy” following other literature on macular pigment measurement.

(line 66) The phrase, ‘local MPODs at 0.25…’ should be attached to the first sentence, because this is the method of measurement.

- Thank you for your suggestion. We revised accordingly.

(line 73) The word, ‘qualities’ should be amended to ‘quality images’.

- Thank you for your suggestion. We revised accordingly.

(line 94) The reference (4) should be placed correctly.

- Thank you for your suggestion. We revised accordingly.

(line 95) For general readers, the authors should describe the details of basic research and clinical studies (, exemplified like ‘to investigate potential for disease prophylaxis and assess pigment density in individuals’).

- Thank you for your suggestion. We revised the description from line 92 to 103.

(line 103) The term, ‘fluorescence of lipofuscin’ should be ‘autofluorescence derived from lipofuscin’. Also, ‘ the retina’ should be ‘retinal pigment epithelial cells’.

- Thank you for your suggestion. We revised accordingly.

(line 121) The authors stated that the blue excitation light is absorbed by the colored lens. How is the influence of yellow-tinted IOL? Also, the autofluorescence of the colored lens may interfere with captured images of fundus autofluorescence. The authors should discuss these issues.

- Thank you for your suggestion. In the case of yellow-tinted intraocular lens, the color of the lens can be considered uniform. However, in the case of nuclear cataracts, the color intensifies toward the center of the lens, and color is not entirely uniform. Since MPOD is calculated based on the ratio of blue light blockage between the targeted lesion and background lesion in autofluorescence spectroscopy, the accurate measurements of MP cannot be achieved in nuclear cataract. But we believe that MP can be measured accurately in eyes with intraocular lens implantation, irrespective of its color.

(line 148) It would be better if the authors could discuss not only the effectiveness of further validation but also the relationship of macular pigments to age and cataract status.

- Thank you for your suggestion. We have addressed the following themes in our previous papers: MP in aged Japanese population (Obana A et al. JJO 2020), grade of cataract and its influence on MP measurement (Obana A et al. IOVS2018), and MP changes after clear IOL implantation and yellow-tinted IOL implantation (Obana A at al. Opthalmol 2011, Obana A et al. PlosOne 2021). We would like to refrain from addressing these themes in this report as they are out of focus of the present report.

(line 211) Is ‘(true value- nominal value) / true value’ right?

- We appreciate your notification. We defined the error in MPOD as follows; error = (MPOD_before_ – MPOD_after_) /MPOD_after_. Since MPOD_after_ is larger than MPOD_before_, the error is expressed as a negative value and we used absolute values in analyses.

(lines 216 and 231; and Table 5) The numbers of high or moderate quality images and poor quality image should be shown. Was there difference in cataract status between these groups? The authors should discuss possible causes of poor qualities. It would be better if the authors could provide representative images.

- Thank you for your suggestions. The examples of three qualities images (high, moderate, and poor) were shown in figure 1 of our previous report. We added the following description in line 302, “Refer to Figure 1 in our previous paper”. We thought that we should avoid to put the images used in our previous paper as they would be redundant for this paper.
- We described the number of high and moderated quality images in line 311. We added the number of these images in total 312 eyes. Demographics of patients with acceptable autofluorescence images and nonacceptable images were shown in Table 1 in our previous study. But we did not evaluate cataract status using the same anterior camera with our previous study because the relationship between image quality and cataract status was not the theme of the present study.

(line 220) It would be better if the authors could explain the details of some exceptions.

- Thank you for your suggestion. The number of eyes that were overestimated before surgery was described in line 234 – 236.

Dear Reviewer #2

We appreciate your review work and thanks for your very useful comments and suggestions on improving our manuscript. We investigated each suggestion carefully and revised our manuscript accordingly. Our reply is written in blue font. The revised parts were highlighted with red font in the revised manuscript.

Reviewer #2: This paper is interesting but has a number of challenges that I have outlined below.
The objective of the study is to determine whether an algorithmic correction can improve MP measures made using the AF technique. The issue, according to the authors, is that AF is confounded by cataracts and alters MP values as a result. The problem is that a dense anterior lens absorbs (and scatters) the measuring light (460 nm) and even some of the reference leading to an overall reduction in MP values. The “smart” correction the authors apply is one that, they argue, can most accurately adjust the MP value back to its true value.

1. One problem in the study (and in previous studies) is that in order to validate this correction, the authors would need to know what the “true” value of MP actually is (not “defined” as true, but actually true). If you are trying to validate a method, it needs to be compared against a known standard. The best way to do this would have been to measure the lens and MP density in adults with healthy eyes (even younger people have large variation in lens density) using validated methods (Like HFP for MP) and then see how the method/corrections works in this best scenario.

- Thank you very much for your highly important remarks. As indicated in our previous papers, measurements of MPOD using autofluorescence spectroscopy result in underestimation, even in cases with mild cataracts. Therefore, our objective is to find a method to correct for the influence of cataracts when measuring MPOD with autofluorescence spectroscopy.
- MPOD measurement by Spectralis was validated in the previous papers of several authors (described in line 125-129), and the focus of the current study is not to validate whether the measurements using Spectralis represent the true MPOD, but to validate if the correction method using AI successfully eliminates the influence of cataracts. However, as you pointed, it is important to validate whether MPOD measured after operation represents the true MP amount. We want to study the theme by comparing HFP and Spectralis in pseudophakic eyes in a future study.
- In our previous papers, we described the postcataract surgery MPOD values as “true values”, but indeed, this was an error as you pointed. The correct terminology is “MPOD after surgery”. We have discontinued the use of the term “true value” and, as outlined in the method (line 214-220), revised the terminology to “MPOD_before_”, “MPOD_after_”, “MPOD_corrected_” to accurately reflect the different stages of measurements.

2. Why is deep learning (or other corrections) better than simply measuring the density and scattering of the lens at the wavelengths used to measure MP? Even if that is too laborious, wouldn’t you want to do this at least for the purpose of checking the accuracy of your derivations?

- Thank you very much for your suggestion. Clinically, as far as we know, there is no method to measure light transmittance and scattering intensity of the crystalline lens. The degree of opacification and the coloration of the lens nucleus vary widely in cataract cases, and the extent of light transmittance and scattering differs from case to case. Therefore, we consider it practical and clinically applicable to utilize AI to assess autofluorescence images and develop a method that eliminates the influence of cataracts,
- We believe that the AI-based approach we propose is a clinically user-friendly method.

3. How do you know it is only the lens confounding AF measures? AF relies on a measuring substrate (lipofuscin) that changes with age and often declines sharply with progressing retinal disease.

- Thank you very much for your suggestion. As you pointed, it is necessary to assume a uniform distribution of lipofuscin to achieve accurate MPOD measurements with autofluorescence spectroscopy. However, the dual-wavelength autofluorescence spectroscopy, which was used in this study, can correct for some ununiform distribution of lipofuscin by employing a green laser in addition to blue excitation laser. Furthermore, the subjects in this study were confirmed not to have any abnormalities in the macula by fundus examination and OCT testing. Therefore, we believe that the MPOD measurements in this study were not influenced by abnormalities in the retina. Additionally, the retinal condition remains the same in both pre-operative and post-operative measurements, therefore, we think. that the images were captured under identical conditions regarding lipofuscin distribution.

4. The original AF method required that the retina is bleached. Was this done? Doesn’t a cataractous lens effect the completeness of the bleach? How do subjects fixate when making peripheral measures if their retina is bleached and they cannot see?

- Thank you for your suggestion. Yes, Spectralis-MPOD bleaches retinal photopigment before measuring MPOD. As you are concerned, there is a possibility that cataracts may affect the bleaching of the retina. This also complicates the accurate measurement of MOPD in the eye with cataracts. However, the current Spectralis program cannot address this issue.
- MPOD measurement with Spectralis follows the same procedure as taking fundus photographs, and patients do not need to gaze at a fixation light, unlike in the case of HFP.

5. The MPOD values in Table 2 seem unrealistic. First, the spatial distribution of values seems very flat (MP is much more leptokurtic). Second, the AF measurement is not at peak MPOD, it is at 486 nm. Your measure at 0.5 degrees (analogous to the standard 1 degree diameter), after surgery, was 0.74. Compare this to Ciulla et al. (2001, IOVS) who also measured MPOD before and after cataract (it is good scholarship to actually cite studies that do the same work). In their group of patients, MPOD was 0.206 before and 0.18 after cataract extraction.

- Thank you for providing a valuable reference. We have cited it and added a note stating that there is no impact of cataracts in HFP (line 130).
- Thank you for your suggestions. Concerning the distribution of MP, we reported previously in Japanese population (Obana A et al. Jpn J Ophthalmol, 62:160-170, 2020). The mean MPOD values from the center of the fovea (0°) to the periphery (9°) were shown in the report (shown below), and we would appreciate it if you could refer to the report. The Spectralis MPOD measurement program incorporates coefficients to correct for the difference between the excitation wavelength and the maximum absorption wavelength of MP. Therefore, our measurements represent values corrected for the difference in wavelength.

- The MPOD values in this study do not significantly differ from those in our previous studies. It is known that there are racial differences in the amount of macular pigment, with reports indicating that Asians have higher pigment levels than Caucasians. As your concern, MPOD at 0.5° eccentricity was reported 0.206 by Ciulla TA et al. In their study, MPOD was measured by HFP with 6° eccentricity as a background. MPOD values vary according to different protocol; for example, Akuffo et al. reported MPOD values ranging from 0.48 to 0.56 at 0.23° eccentricity. They used the same instrument with us but set 7° eccentricity as for background. One reason for higher MPOD in our report is the setting of background. We set 9° eccentricity as for background. To eliminate differences in measurement protocol, we calculated MPOD values under the same conditions as reported by Green-Gomes et al, although it is necessary to note that a different Spectralis machine was used. As a result, in the Green-Gomes report, MPOV (total volume of MP) was 4,729 for Irish individuals, 8,307 for Mexicans, and in our study, 10,010 for Japanese. Incidentally, the value for individuals who underwent cataract surgery with intraocular lens implantation was 12,135. Based on these results, we think that the macular pigment levels in Japanese individuals are higher than those in Caucasians.
