# Supplementary material for: Deep learning-based correction of cataract-induced influence on macular pigment optical density measurement by autofluorescence spectroscopy
Source: PLoS One. 2024 Feb 13;19(2):e0298132. doi: 10.1371/journal.pone.0298132 (PMC10863874; doi:10.1371/journal.pone.0298132)
Supplement: Attachment — Submitted filename: renamed_815ce.docx [file pone.0298132.s004.docx]

Reply to reviewers.

Dear Reviewer #2

Thank you so much for conducting the second review. However, we regret that our responses to the first review did not seem to obtain your full understanding. What we are seeking is a relatively simple method to measure MP which can be used in outpatient ophthalmic care. Spectralis is very widely used as an OCT examination, and measuring MP with Spectralis is very convenient for ophthalmologists. However, as discussed in the previous reports and this paper, Spectralis has the drawback that excitation light is affected by cataracts.

In our previous report, we reported that using AI to assess the image quality of autofluorescence photographs for calculating MP can reduce measurement errors. However, since the previous report only validated the method among internal data, in this study, we validated this approach using external data. The main point of this paper lies in proving the validity of the correction method using AI through external validation. The purpose of this paper is not to study the value of MP, factors involved in MP, or the function of MP. These aspects have been reported by many researchers, including in our previous work.

Taking the above into consideration, I will respond to your criticisms.

1. In the revisions, the authors write "...The macular pigment (MP) absorbs short-wavelength 93 visible light and works to filter blue light. Blue light is highly scattered in the retina... The MP absorbs short-wavelength light AND works to filter blue light? Those are basically the same thing. Also, blue light is mostly scattered in the anterior media (the lens) not the retina. These kind of small technical errors are pretty pervasive.

Thank you for your comment, but we respectfully disagree with you.

UV-B and C do not transmit to the retina, but blue visible light definitely transmits to the retina and is captured by the blue-sensitive cones. Blue light is more prone to scattering within tissues compared to longer-wavelength light, and MP suppresses scattered light within the retina. It has been reported that individuals with higher amount of MP exhibit higher contrast sensitivity (Nolan JM. IOVS 2016;57:3429), and the reason for this is supposed to be the suppression of scattered light by MP. However, as you pointed out, blue visible light scatters in the corneal opacity and cataract, so we have revised the description in line 92-93. We apologize for any misunderstanding caused by our initial description.

2. In your response letter, you write "...Clinically, as far as we know, there is no method to measure light transmittance and scattering intensity of the crystalline lens." There are many fairly easy methods for measuring the density and scattering of the lens. Further, I am not sure why the method would need to be based on a clinical instrument. This is a research study. You can use more elaborate methods first and then if the method is valid it can be translated for use within the clinic.

In the clinical setting of Ophthalmology, the severity of cataracts is determined by ophthalmologist through the observation of the lens using a slit lamp, following international grading system (Thylefors B. Ophthalmic Epidemiol 2002;9:83). This subjective assessment method is also utilized in many clinical studies. Although our previous report utilized a Scheimpflug camera, it is still a subjective judgement method. If there were a device that could be applied to human and safely measure the light transmission of the crystallin lens, it would be possible to correct the measurement errors of MP based on light transmission. This would be an ideal correction method, as you suggested. However, to the best of our knowledge, such a device has not been commercially available, and hence we have to seek for a method to measure MP that can be readily used at clinical practice rather than in basic research. Therefore, we explored a method of using AI to assess the image quality of autofluorescence photographs. If you are aware of a method for measuring the light transmission of the crystallin lens that can be used in clinical practice, we would appreciate it if you could share the information with us. We want to apply it in our next study.

Dear Reviewer #3

We appreciate your review work and thanks for your positive comments.

Regarding the instruction to add a boxplot, in the previous report, boxplots related MP before and after cataract surgery were included. Although it is possible to create similar boxplots with the data from this paper, we have chosen to omit them as they would be highly redundant. We appreciate your understanding.
